# Relationship between the extent of resection and the survival of patients with low-grade gliomas: a systematic review and meta-analysis

**DOI:** 10.1186/s12885-017-3909-x

**Published:** 2018-01-06

**Authors:** Liang Xia, Chenyan Fang, Gao Chen, Caixing Sun

**Affiliations:** 1 Department of Neurosurgery, Zhejiang Cancer Hospital, 1 ban shan east Road, Hangzhou, Zhejiang Province 310022 China; 2 Department of Neurosurgery, The second affiliated hospital of Zhejiang University, Hangzhou, Zhejiang Province 310000 China; 3 Zhejiang Cancer Hospital, Zhejiang Chinese medical university, Hangzhou, Zhejiang Province 210022 China

**Keywords:** Extent of resection, Low-grade Gliomas, Prognosis

## Abstract

**Background:**

Surgical resection is necessary to conduct a pathological biopsy and to achieve a reduction of intracranial pressure in low-grade gliomas patients. This study aimed to determine whether a greater extent of resection would increase the overall 5-year and 10-year survival of patients with low-grade gliomas.

**Methods:**

The studies addressing relationship between the extent of resection and the prognosis of low-grade gliomas updated until March 2017 were systematically searched in two databases (Pubmed and EMBASE). The relationships among categorical variables were analyzed using an odds ratio (OR) and a95% confidence interval (CI). Significance was established using CIs at a level of 95% or *P* < 0.05. Funnel plot was used to detect the publication bias.

**Results:**

Twenty articles (a total of 2128 patients) were identified. The meta-analysis showed that the 5-year (Odds ratio (OR), 3.90;95% Confidence Interval (CI), 2.79~5.45; *P* < 0.01; Z = 7.95) and 10-year OS (OR, 7.91; 95%CI, 5.12~12.22; *P* < 0.01; Z = 9.33) associated with gross total resection (GTR) were higher than those associated with subtotal resection (STR). Similarly, as compared with biopsy(BX), the 5-year and 10-year OS were higher after either GTR (5-year: OR, 5.43; 95%CI, 3.57~8.26; *P* < 0.01; Z = Z = 7.9; 10-year: OR, 10.17; 95%CI, 4.02~25.71; *P* < 0.00001; Z = 4.9) or STR (5-year: OR, 2.59; 95%CI, 1.81~ − 3.71; *P* < 0.00001; Z = 5.19; 10-year: OR, 2.21; 95%CI, 1.164.25; *P* = 0.02; Z = 2.39).

**Conclusions:**

Our research found that a greater extent of resection could significantly increase the OS of patients with low-grade gliomas.

**Electronic supplementary material:**

The online version of this article (10.1186/s12885-017-3909-x) contains supplementary material, which is available to authorized users.

## Background

Low-grade gliomas include astrocytoma, oligodendrogliomaand oligoastrocytoma of WHO gradeI-II [1]. The incidence of low-grade gliomas is significantly lower than that of high-grade glioblastomas of all primary intracranial tumors [2]. The epidemiological features, clinical manifestations, proliferation rates, mitotic counts, as well as angiogenesis and genetic features of low-grade gliomas are different from those of high-grade gliomas [3]. Low-grade gliomas have a better prognosis than high-grade gliomas. The established risk factors influencing the prognosis of high-grade gliomas include IDH mutation, age, KPS score, and the extent of resection [4]. However, the prognostic factors of low-grade gliomas are not fully elucidated yet. So far, only IDH mutation, KPS score, age and the pathological type are recognized as factors related to the prognosis of low-grade gliomas [5], the effect on prognosis of extent of resection of low-grade gliomas has not been systematically evaluated.

Many neurosurgeons recommend performing the greatest extent of resection safely possible for both high-grade and low-grade gliomas. In the past, the available surgical techniques might make it difficult to access gliomas in deep locations or in the brain functional areas [6]. However, with the use of neuronavigator and intraoperative MRI, many difficulties in accessing the tumors in challenging locations been solved [7]. High-grade gliomas have a higher incidence and their median survival ranges from 1 to 2 years [4]. A large number of researches have tried to elucidate the association between the extent of resection and the prognosis of high-grade gliomas. There are explicit first-level evidences indicating that a gross total resection (GTR) can significantly increase the overall survival (OS) and progression-free survival (PFS) of patients as compared with a subtotal resection (STR) or biopsy(BX) [8]. In contrast, low-grade gliomas have a lower incidence and a better prognosis. The medial survival of patients with low-grade gliomas is 5 to 10 years [3]. However, there are much fewer cases of low-grade gliomas and relevant clinical trials are hindered by long trial durations and ethical principles. At present, few researches have been focused on the relationship between the extent of resection and the prognosis of low-grade gliomas. Therefore, no randomized trials or first-level evidence have demonstrated explicitly the relationship between the extent of resection and prognosis.

It remains controversial whether a greater extent of resection can increase the OS and PFS of patients with low-grade gliomas. We performed a meta-analysis to prove the relationship between the extent of resection and the prognosis of patients with low-grade gliomas, then provide a basis for the development of evidence-based medicines in low-grade gliomas.

## Methods

### Search strategy and study selection

Using the PICO strategy, PubMed and EMBASE were searched for publications up to March 2017. The keywords chosen for the search included low-grade glioma (WHO gradeI-II), extent of resection, resection, biopsy and survival. The scope of search was expanded by combining the keywords with non-keywords according to the restriction of the English-language. Auxiliary search techniques such as keywords expansion were used to increase the recall rate. Detailed search strategies for both databases are shown in Additional file 1: Appendix 1.

Search was performed according to the above strategy to obtain the titles of relevant articles. Subsequently, the search strategy was adjusted based on the number of articles obtained after the preliminary search. Articles obtained in this manner were further screened. For systemic reviews related to this topic, their bibliographies were searched to identify potential articles.

All included articles were reviewed by two independent reviewers (Xia L and Fang CY). All disagreements were settled through discussion. If the disagreements could not be settled, a third party was invited to make a final decision. The eligible criteria were as follows: (1) Patients with low-grade gliomas diagnosed by pathology; (2) Adult patients with lesions in the supratentorial region; (3) Trials discussing the relationship between the extent of resection (GTR, STR or biopsy) and prognosis (OS or PFS); (4) 5-year or 1-year OS data were available or could be calculated from other results such as survival plots; (5) If the included cases overlapped, the trial with a greater number of cases would be included. Exclusion criteria were as follows: (1) Patients were pathologically diagnosed as high-grade gliomas in most of the cases included in the article; (2) Patients with pediatric gliomas or subtentorial gliomas; (3) The extent of resection was expressed as percentages rather than GTR, STR and biopsy; (4) 5-year or 10-year survival data were not available.

### Data extraction

Low-grade gliomas are associated with a better prognosis and only a few studies have been focused on the 1-year or 2-year survival of patients with low-grade gliomas. The literature search also yielded a limited number of studies covering this topic. Therefore, the topic in the search was changed to the association between the extent of resection and 5-year and 10-year OS of patients with low-grade gliomas. Data extraction was performed by two independent reviewers, and the data included the name of the first author, publication time, country, sample size, patients’ age, tumor type, the extent of resection, 5-year or 10-year OS and the duration of follow-up. The extent of resection was divided into three categories, i.e., GTR, STR and BX. STR included both subtotal resection and partial resection. If the survival rate was not mentioned when endpoints were reached, the survival rate was calculated from the Kaplan-Meier curve. Already mentioned that disagreements were settled with discussion.

### Quality assessment of primary studies

The quality of each article was assessed using American Academy of Neurology level of evidence criteria by a research team with four members. All included articles were scored independently by four members and then the sum score was obtained. Any disagreement was settled through discussion until a consensus was reached. The included articles were classified into level I-IV. Among them, Level I indicated the best quality while level IV indicated the lowest credibility.

After data sorting and meta-analysis, the credibility of evidence was assessed using the GRADE system. There were four levels of credibility, i.e., high, moderate, low and very low. A high quality was assigned if the outcome assessment could be altered by further studies; a moderate quality was assigned if the credibility of the outcome assessment and the outcome assessment itself might be altered by further studies; a moderate quality was assigned if the credibility of the outcome assessment and the outcome assessment itself might be altered by further studies; a very low quality was assigned if any outcome assessment was uncertain.

### Statistical analysis

Revman5.3 software provided by Cochrane collaboration was employed. Depending on the forest plot and results from the tests of heterogeneity, a fixed effects model or a random effects model was chosen. The relationships among categorical variables were analyzed using an odds ratio (OR) and a95% confidence interval (CI). Significance was established using CIs at a level of 95% or *P* < 0.05.Logarithmicdata were processed by weighting on the basis of sample size. That is, the greater the sample size, the greater the weight was assigned. Funnel plot was used to detect the publication bias.

## Results

### Literature search

**Fig. 1:**
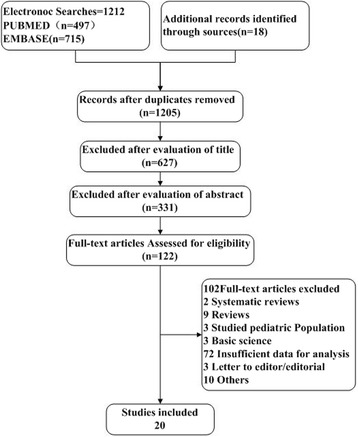
Flowchart of study selection

According to the search strategy, a total of 1230 English articles (Fig. 1) were eligible. After reviewing the titles, abstracts and full texts, 1210 articles were excluded. Finally, 20 articles involving 2128 cases were included for the meta-analysis (one was a randomized and controlled trial (RCT) and 19 were retrospective studies).

### Article quality assessment

None of the included articles was a class Level I study. There were 4 [9–12] class Level II studies, 13 [13–25] class Level III studies and 3 [26–28] class Level IV studies (Table 1). Only 1 study involved a prospective RCT.

**Table 1:** Included study characteristics

Study	Publication Time	Years of Patient Accrual	Country	Study Type	No. of Patients	No. of Analysis	Median Follow Up	Histology	Adjuvant Therapies	EOR assessment	Level of Evidence
WINGE(9)	1989	1982–1987	London	retrospective study	285	187	NS	Glioblastoma multiforme 188 Astrocytoma 76 Mixed 11 Oligodendroglioma 10 Astrocytoma or mixed A > O 32	RT	NS	II
Shaw(10)	2002	1986–1994	Mayo	retrospective study	203	203	6.4 years	Oligodendroglioma or mixed O > A 69	RT	NS	II
HARMON J(11)	1993	1980–1985	USA (Michigan)	prospective randomized trial	60	60	4.5 years	Low-grade Glioma	RT + Ch	NS	II
Edward G(12)	1994	1960–1982	Mayo	retrospective study	71	71	15 years	Oligoastrocytoma	RT	operative report	II
R. Laws(13)	1984	1915–1975	Mayo	retrospective study	851	461	15 year MAX	Astrocytoma	RT	microscopically	III
LINDEGAARD(14)	1987	1953–1977	Norway	retrospective study	58	58	23 years MAX	Oligodendroglioma	RT	NS	III
SHAW(a)(15)	1989	1915–1975	Mayo	retrospective study	41	41	25 years MAX	Astrocytoma	RT	operative report	III
Soffietti(16)	1989	1950–1982	Italy	retrospective study	71	71	7 year MAX	Astrocytoma	RT	Surgeon’s description	III
North(17)	1990	1975–1984	USA(Baltimore)	retrospective study	32	32	69mo	Astrocytoma	RT	NS	III
SHAW(18)	1992	1960–1982	Mayo	retrospective study	82	82	7.1 years	Oligodendroglioma	RT	operative report	III
Shibamofo(19)	1993	1965–1989	Japan (Kyoto)	retrospective study	67	67	10 year MAX	Astrocytoma	RT + Ch	NS	III
Paolo(20)	1994	1953–1986	Italy	retrospective study	105	105	NS	Oligodendroglioma	RT	operative report	III
RAJAN(21)	1994	1974–1990	UK	retrospective study	82	82	52mo	Glioma	RT	operative report	III
Veelen(22)	1998	1975–1989	Netherlands	retrospective study	90	90	120mo Max	Astro 72 Oligo 14 Mixed 4	RT	patients’ charts	III
IWABUCHI(23)	1999	1967–1993	Japan	retrospective study	56	56	5 year	Astrocytoma	RT	NS	III
Smith(24)	2008	1989–2005	USA (California)	retrospective study	216	216	4.4 years	Astrocytoma 93 Oligodendroglioma 91 Mixed Oligoastrocytoma 32	RT + Ch	Patientrecords	III
Thomas B. Daniels(25)	2012	1984–2007	Japan	retrospective study	42	42	NS	Astrocytoma 27 Oligodendroglioma12 Oligoastrocytoma7	RT + Ch	Pre- and post-operative CT	III
Nicolato(26)	1995	1977–1989	Italy	retrospective study	76	74	160mo Max	Astrocytoma	RT	NS	IV
Jeremica(27)	1998	1988–1993	Japan	retrospective study	37	37	74mo	Astrocytoma 18 Oligodendroglioma 14 Mixed glioma 5	RT	operative report	IV
YEH(28)	2005	1985–1997	China (Taiwan)	retrospective study	93	93	110mo	Astrocytoma 46 Oligodendroglioma 32 Mixed 15	RT	operative report	IV

Note: *NS* Not stated, *RT* Radiation therapy, *Ch* chemotherapy not otherwise specified

### Publication bias

A funnel plot was used to detect the bias in the above articles (Fig. 1). The data points were all located inside the inverted funnel, indicating a small publication bias.

### Quality for the body of evidence (GRADE rating)

The GRADE rating was performed to assess the quality of evidence in terms of the OS outcome and it was found that the quality was of a moderate level. The quality of evidence in class 2 studies was also moderate. The quality of evidence in other studies was low.

### Meta-analyses for five-year survival rates

**Fig. 2:**
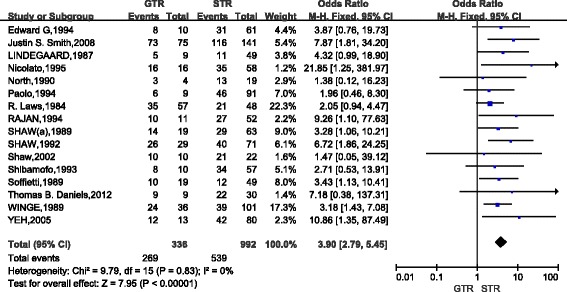
Forest Plot of 5-Year Overall Survival for Gross Total Resection (GTR) vs Subtotal Resection (STR)

**Fig. 3:**
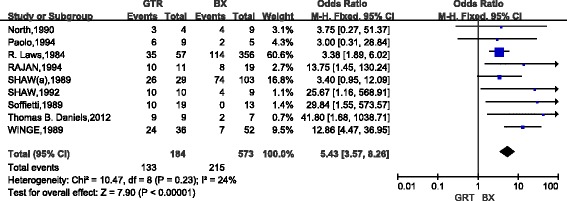
Forest Plot of5-Year Overall Survival for Gross Total Resection (GTR) vs Biopsy (BX)

**Fig. 4:**
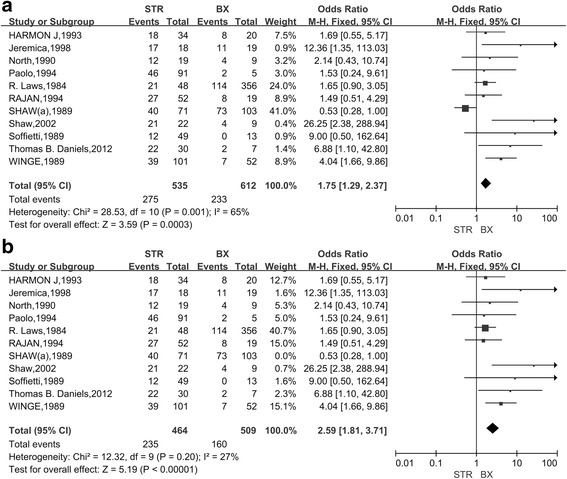
Forest Plot of 5-Year Overall Survival for Subtotal Resection (STR) vs Biopsy (BX)A. All related studies were included; B. All related studies except one high heterogeneous study were included

Among the 20 included studies, 16 studies (1328 cases) compared the 5-year OS of patients with low-grade gliomas after GTR and STR (Fig. 2). The combined results indicated that, as compared with STR, GTR could significantly increase the 5-year survival of patients with low-grade gliomas (OR, 3.90; 95%CI,2.79~5.45) and there was nearly no heterogeneity between studies (*P* = 0.83). Nine studies (775 cases) compared the 5-year survival between GTR and biopsy (Fig. 3). The pooled results indicated that, as compared with biopsy, GTR could significantly increase the 5-year survival of patients with low-grade gliomas (OR, 5.43; 95%CI,3.57~8.26) and there was nearly no heterogeneity between studies (*P* = 0.23). Eleven studies (1147 cases) compared the 5-year survival of patients with low-grade gliomas between STR group and BX group. The combined results indicated that, as compared with BX, STR significantly increased the 5-year survival (OR,1.75; 95%CI,1.29~2.37) but the heterogeneity was high (I2 = 65%, *P* = 0.001)(Fig. 4a).Based on the funnel plot, one study was excluded due to high heterogeneity. Analysis of the remaining 10 studies further proved that STR increased the 5-year survival as compared with biopsy (OR,2.59; 95%CI, 1.81~3.71; *P* < 0.01; Z = 5.19) (Fig. 4b).

### Meta-analyses for ten-year survival rates

**Fig. 5:**
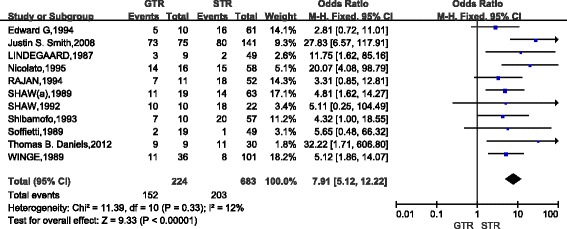
Forest Plot of 10-Year Overall Survival for Gross Total Resection (GTR) vs Subtotal Resection (STR)

**Fig. 6:**
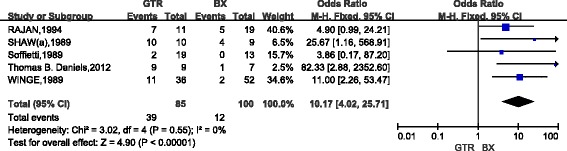
Forest Plot of 10-Year Overall Survival for Gross Total Resection (GTR) vs Biopsy (BX)

**Fig. 7:**
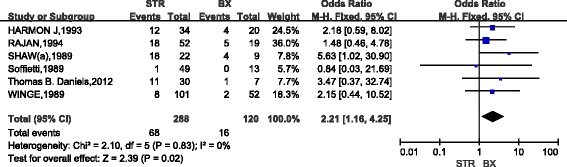
Forest Plot of 10-Year Overall Survival for Subtotal Resection (STR) vs Biopsy (BX)

A total of 11 studies (907 cases) compared the 10-year survival of patients with low-grade gliomas after GTR and STR. The combined results indicated that, as compared with STR, patients with GTR had the poor 10-year survival(OR,7.91; 95%CI,5.12~12.22)and there was no apparent heterogeneity between studies (*P* = 0.33) (Fig. 5). Five studies (185 cases) compared the 10-year survival between GTR and biopsy in low-grade gliomas. The combined results indicated that, as compared with biopsy, GTR considerably increased the 10-year survival (OR,10.17; 95%CI,4.02~25.71) and there was no heterogeneity between studies (*P* = 0.55) (Fig. 6). Six studies (408 cases) compared the 10-year survival in low-grade gliomas after STR and biopsy. The combined results indicated that, as compared with biopsy, STR considerably increased the 10-year survival(OR,2.21; 95%CI,1.16~4.25)and there was also no heterogeneity between studies (*P* = 0.83) (Fig. 7).

### Quality for the body of evidence (GRADE rating)

The GRADE rating was performed to assess the quality of evidence in terms of the OS outcome and it was found that the quality was of a moderate level. The quality of evidence in Level II studies was also moderate. The quality of evidence in other studies was low.

### Publication bias

Funnel plots were used to detect the bias in the above articles (Additional file 2: eFigure S1, Additional file 3: eFigure S2, Additional file 4: eFigure S3, Additional file 5: eFigure S4, Additional file 6: eFigure S5, Additional file 7: eFigure S6). Except studies comparing the 5-year survival of patients with low-grade gliomas between STR group and biopsy group, no publication bias was found in funnel plots, with plots visually symmetrically distributed along the vertical axis.

## Discussion

The clinical value of surgery in low-grade gliomas is heavily disputed [29]. Researchers suggest that although surgery is conducive to pathological diagnosis and remission of symptoms, some low-grade gliomas show infiltrative growth and it is difficult to achieve radical cure through a simple surgery [30]. Low-grade gliomas are generally located in the brain functional areas with obscure boundaries. Surgical resection of low-grade gliomas may lead to dysfunction and impairment of patients’ quality of life (QOL) [31]. Some reports showed that the 5-year and 10-year survival of patients receiving GTR was comparable to those receiving STR or no surgery at all [32–34].For this reason, GTR is not the first-line therapy for low-grade gliomas. In recent years, there has been a trend of favoring GTR in the treatment of low-grade gliomas [35, 36].Therefore, we reviewed relevant studies published up to 2017 and performed a quantitative meta-analysis. The results showed that GTR greatly increased the 5-year and 10-year survival of patients with low-grade gliomas.

Meta-analysis can enhance the credibility of conclusions by using a larger sample size and therefore can resolve the inconsistency among different studies [37]. The findings of meta-analysis are more reliable than those of a single study [38]. Twenty studies focusing on the surgical outcomes of low-grade gliomas were analyzed, as the result showed in the Table 2, patients with GTR had better prognosis than those with STR and biopsy, similarly, STR is superior to biopsy both in the 5-year and 10-year OS. Thus, patients with low-grade gliomas are expected to benefit from a greater extent of resection if their safety during the surgery can be ensured.

**Table 2:** Subgroup meta-analysis results

Study Factors	NO. of Included Studies	NO. of Patients	Survival Rate	Odds Ratio M-H,Fixed (95%CI)	*P*-value	I^2^statistics, *P*-value
Five-Year Overall Survival						
GTR	16	336	80.06%	3.90 [2.79,5.45]	P< 0.00001	0%, 0.83
STR		992	54.33%			
GTR	9	184	72.28%	5.43 [3.57, 8.26]	P<0.00001	24%, 0.21
BX		573	37.52%			
STR	10	464	50.65%	2.59 [1.81, 3.71]	P< 0.00001	27%, 0.20
BX		509	31.43%			
Five-Year Overall Survival						
GTR	11	224	67.86%	7.91[5.12,12.22]	P< 0.00001	12%, 0.33
STR		683	29.72%			
GTR	5	85	45.88%	10.17[4.02,25.71]	P< 0.00001	0%, 0.55
BX		100	12%			
STR	6	228	29.82%	2.21[1.16,4.25]	*P* = 0.020	0%, 0.83
BX		120	13.33%			

Better outcome following GTR can be explained by the types of growth that low-grade gliomas exhibit. Firstly, the growth of low-grade gliomas can be divided into three types: confined growth, invasive growth and malignant change. According to recent studies, low-grade gliomas show continuous and slow confined growth before malignant change, resulting in an annual increase of about 4 mm in size [30]. Invasive growth of low-grade gliomas is demonstrated as the invasion of adjacent white matter tracts, or even the invasion into the contralateral side via corpus callosum. In addition, low-grade gliomas may evolve into high-grade gliomas [39]. It was reported that 66.4% of patients with low-grade glioma sunder went de-differentiation within 5 years after surgery, resulting in a worse prognosis [40]. Therefore, early resection of tumor is very important to control infiltration and metastasis. Moreover, the reduction in the tumor load is also conducive to improve effectiveness of subsequent radiochemotherapy. Secondly, from the pathological aspect, histopathology remains the gold standard for the malignancy classification of gliomas. The accuracy of histopathological diagnosis depends on whether a submitted sample is representative [41]. Gliomas are usually associated with heterogeneity in terms of the varying types of cells and different degrees of malignancy in the tumor. That is why the representativeness of the submitted sample is crucial for pathological diagnosis [42]. In one study, a pathological diagnosis based on stereotactic biopsy of astrocytoma was compared with the diagnosis obtained from a surgically resected sample. It was found that sterotactic biopsy underestimated the grades of tumors in 10%–25% of the cases [43]. Therefore, an extensive resection of low-grade gliomas and the submission of all resected specimens for pathological examination can reduce diagnostic errors.

However, some studies might have biases due to limited technical skills and defects in their experimental designs [44]. For example, many researches concerning surgical treatment for low-grade gliomas were retrospective studies. In other studies, the extent of resection was determined based on neurosurgeons’ experience or CT scan, which might lead to inconsistent conclusions. Recently, National Cancer Institute (NCI) presented a statistics report on the survival of 2009 patients with low-grade gliomas between 1973 and 2001 [35]. The results showed that surgery prolonged the survival of these patients.

There were other limitations in our study. On the one hand, our meta-analysis included only one prospective and randomized controlled clinical trial while most of the included articles were retrospective studies. There were only four Level II studies included in our analysis while many of the remaining studies were of Level III. However, all results from Level II studies were consistent with our result which favored GTR over STR and biopsy. On the other hand, as mentioned above, low-grade gliomas have a much lower incidence than that of high-grade gliomas, leading to a smaller number of eligible trials in the meta-analysis, so large-scale randomized clinical trials for low-grade gliomas are urgently needed. Additionally, there were some covariates between studies, such as patients’ age, auxiliary treatment methods, tumor size and complications, which could bring some bias to our study.

## Conclusion

Our meta-analysis included only one prospective and randomized controlled clinical trial while most of the included articles were retrospective studies. All evidences were consistent (four class 2 studies) in that they favored GTR over STR and biopsy. There were four class2 studies while many of the remaining studies were of class3. We believe that, as compared with STR and biopsy, GTR could significantly increase the 5-year and 10-year survival of patients with low-grade gliomas. If feasible, GTR is recommended for those patients newly diagnosed as low-grade gliomas. Based on the existing evidences, we believe that more retrospective cohort studies may not provide more useful data. Instead, for low-grade gliomas, high-quality prospective clinical trials are needed to analyze the prognostic factors such as the extent of resection, auxiliary treatment, tumor size, tumor location and complications.

## Additional files

Additional file 1: **Supplementary Appendix 1**. (DOC 28 kb)

Additional file 2: eFigure S1. Funnel plot for the 5-year mortality for GTR vs STR meta-analysis. The midline of the studies indicates a slight publication bias of studies showing benefit with GTR over STR. (PDF 12 kb)

Additional file 3: eFigure S2. Funnel plot for the 5-year mortality for GTR vs BX meta-analysis. The midline of the studies indicates a slight publication bias of studies showing benefit with GTR over STR. (PDF 12 kb)

Additional file 4: eFigure S3A. Funnel plot for the 5-year mortality for STR vs BX meta-analysis. The midline of the studies indicates a slight publication bias of studies showing benefit with GTR over STR (All related studies were included). eFigure3B. Funnel plot for the 5-year mortality for STR vs BX meta-analysis. The midline of the studies indicates a slight publication bias of studies showing benefit with GTR over STR (All related studies except one high heterogeneous study were included). (PDF 2433 kb)

Additional file 5: eFigure S4. Funnel plot for the 10-year mortality for GTR vs STR meta-analysis. The midline of the studies indicates a slight publication bias of studies showing benefit with GTR over STR. (PDF 12 kb)

Additional file 6: eFigure S5. Funnel plot for the 5-year mortality for GTR vs BX meta-analysis. The midline of the studies indicates a slight publication bias of studies showing benefit with GTR over STR. (PDF 12 kb)

Additional file 7: eFigure S6. Funnel plot for the 5-year mortality for STR vs BX meta-analysis. The midline of the studies indicates a slight publication bias of studies showing benefit with GTR over STR. (PDF 12 kb)

## Abbreviations

BX: Biopsy
Ch: Chemotherapy not otherwise specified
CI: Confidence interval
GTR: Gross total resection
NCI: National Cancer Institute
NS: Not stated
OR: Odds ratio
OS: Overall survival
PFS: Progression-free survival
QOL: Quality of life
RCT: Randomized and controlled trial
RT: Radiation therapy
STR: Subtotal resection
