# Supplementary material for: Relationship between the extent of resection and the survival of patients with low-grade gliomas: a systematic review and meta-analysis
Source: BMC Cancer. 2018 Jan 6;18:48. doi: 10.1186/s12885-017-3909-x (PMC5756328; doi:10.1186/s12885-017-3909-x)
Supplement: Supplementary file 1 — Supplementary Appendix 1. (DOC 28 kb) [file 12885_2017_3909_MOESM1_ESM.doc]

**PUBMED SEARCH (1 May 2017)**

**Search (((((low grade glioma[Title/Abstract]) OR gliome de bas grade[Title/Abstract]) OR LGG[Title/Abstract])) AND ((((((Survival[Title/Abstract]) OR mortality[Title/Abstract]) OR Prognosis[Title/Abstract]) OR outcome[Title/Abstract])) OR "Survival"[Mesh])) AND ((((extent of resection[Title/Abstract]) OR degree of resection[Title/Abstract]) OR resection[Title/Abstract]) OR biopsy)**

**Results: 497**

**Embase Session Results (1 May 2017)**

**No. Query Results**

**#8 #6 AND #7 AND #1 715**

**#7 #4 OR #5 2602217**

**#6 #2 OR #3 4183385**

**#5 survival:ab,ti OR mortality:ab,ti OR prognosis:ab,ti OR outcome:ab,ti AND [embase]/lim 2401919**

**#4 'survival'/exp 841886**

**#3 resection:ab,ti OR 'extent of resection':ab,ti OR 'degree of resection':ab,ti AND [embase]/lim 271223**

**#2 'surgery'/exp 4146016**

**#1 'low grade glioma':ab,ti OR 'gliome de bas grade':ab,ti OR 'low level glioma':ab,ti OR lgg:ab,ti AND [embase]/lim 3373**
