# Supplementary figures and images for: Relationship between the extent of resection and the survival of patients with low-grade gliomas: a systematic review and meta-analysis
Source: BMC Cancer. 2018 Jan 6;18:48. doi: 10.1186/s12885-017-3909-x (PMC5756328; doi:10.1186/s12885-017-3909-x)

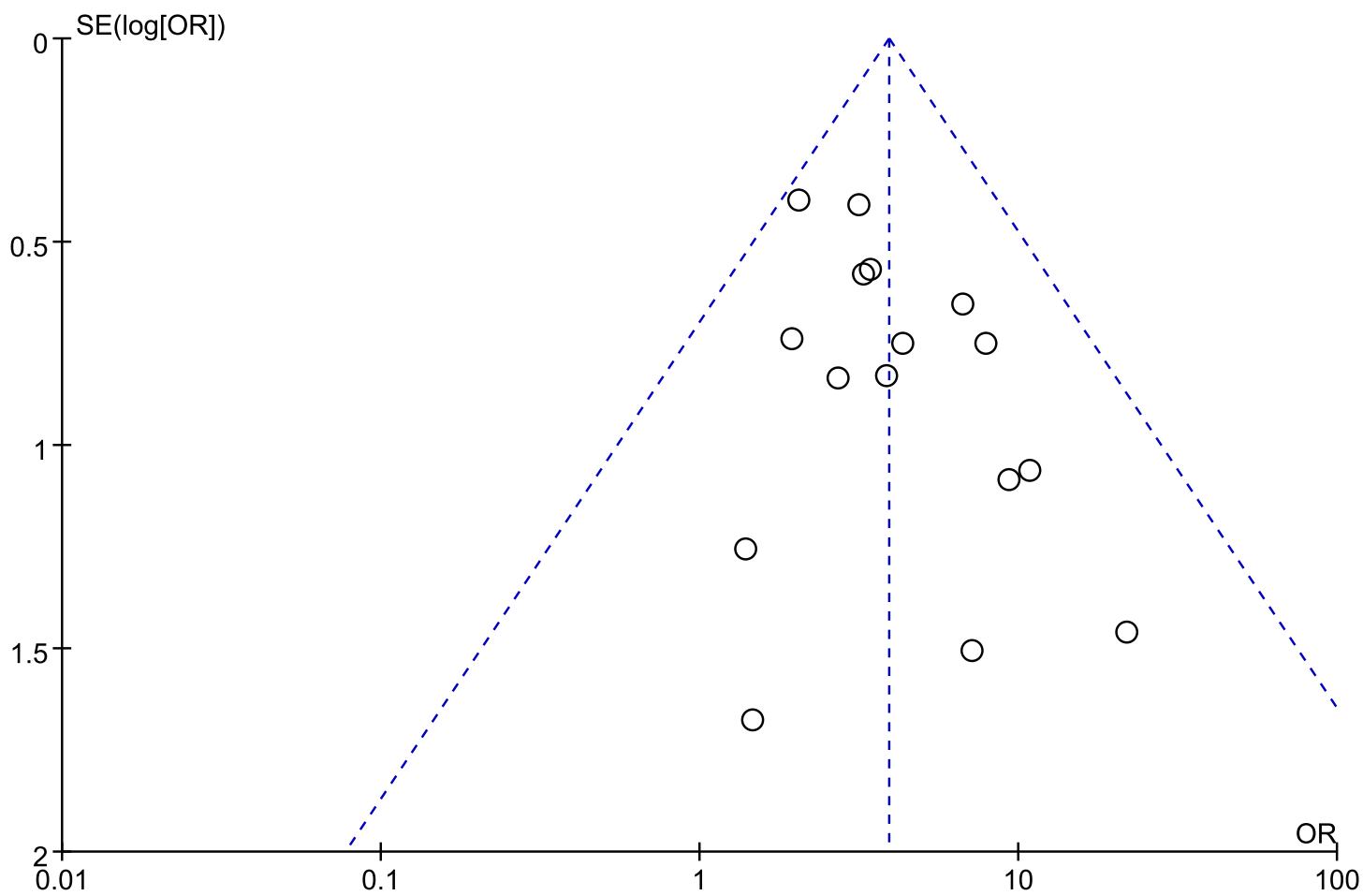

Supplement: Supplementary file 2 — Funnel plot for the 5-year mortality for GTR vs STR meta-analysis. The midline of the studies indicates a slight publication bias of studies showing benefit with GTR over STR. (PDF 12 kb) [file 12885_2017_3909_MOESM2_ESM.pdf]

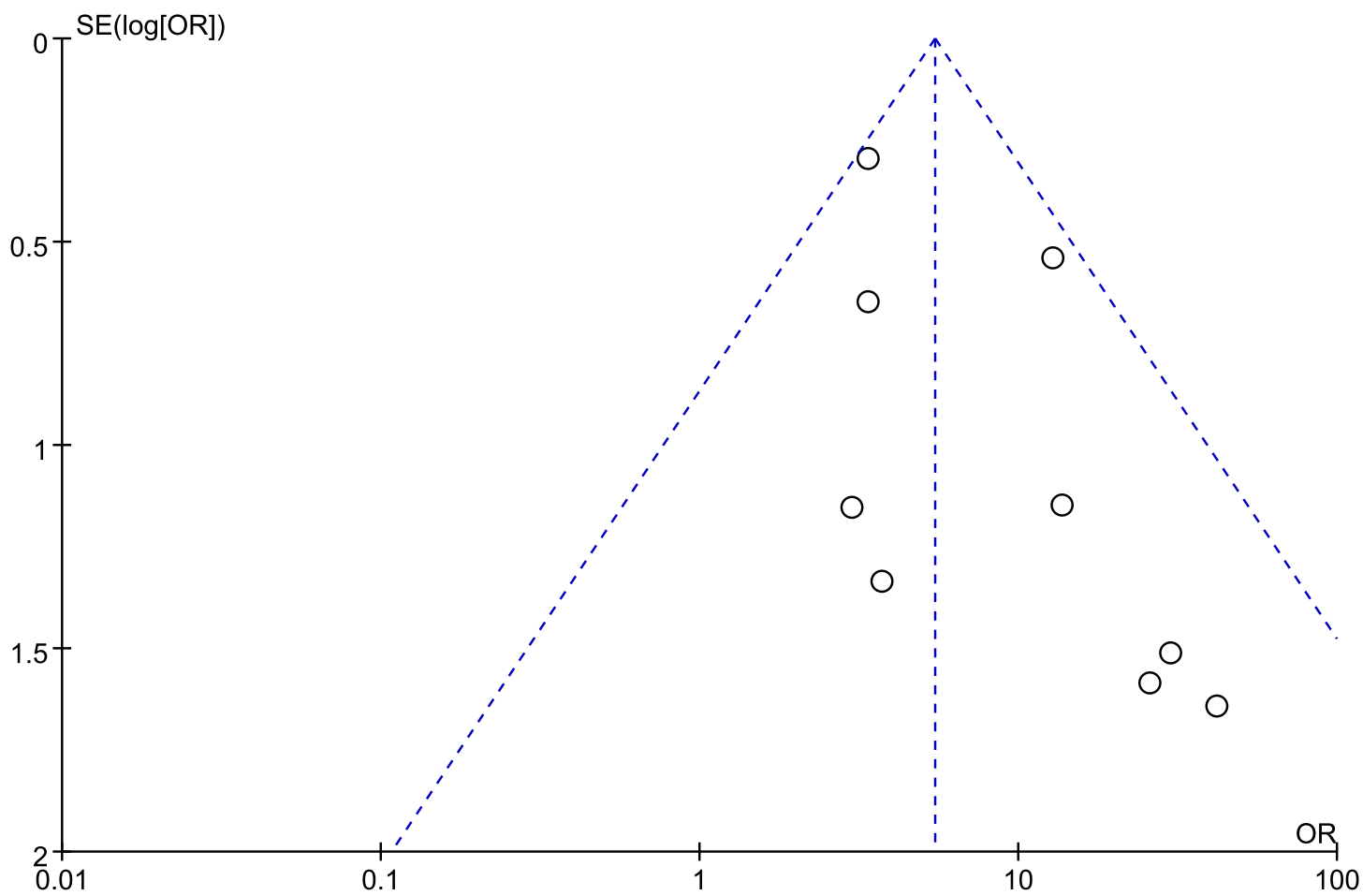

Supplement: Supplementary file 3 — Funnel plot for the 5-year mortality for GTR vs BX meta-analysis. The midline of the studies indicates a slight publication bias of studies showing benefit with GTR over STR. (PDF 12 kb) [file 12885_2017_3909_MOESM3_ESM.pdf]

A

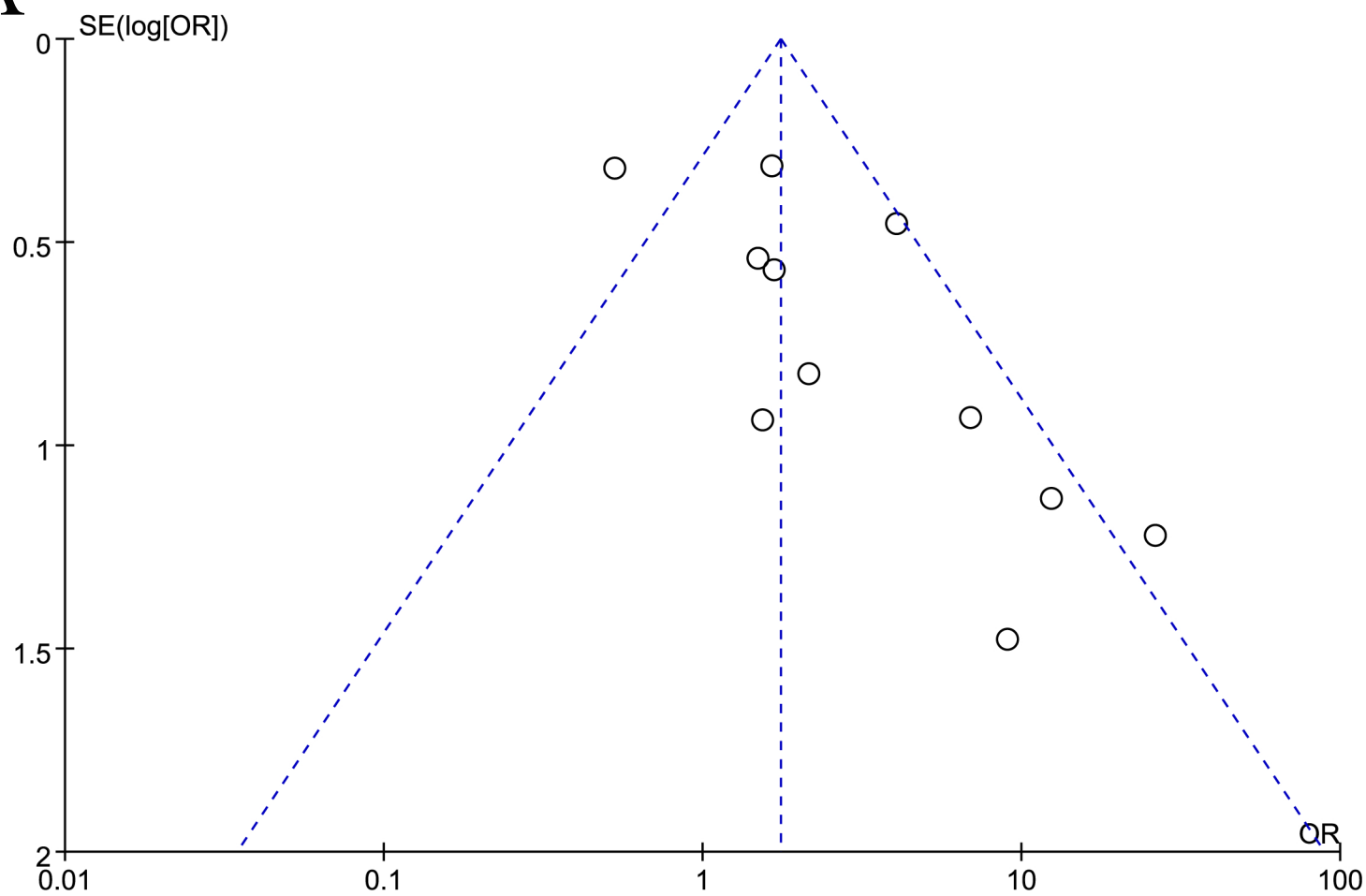

B

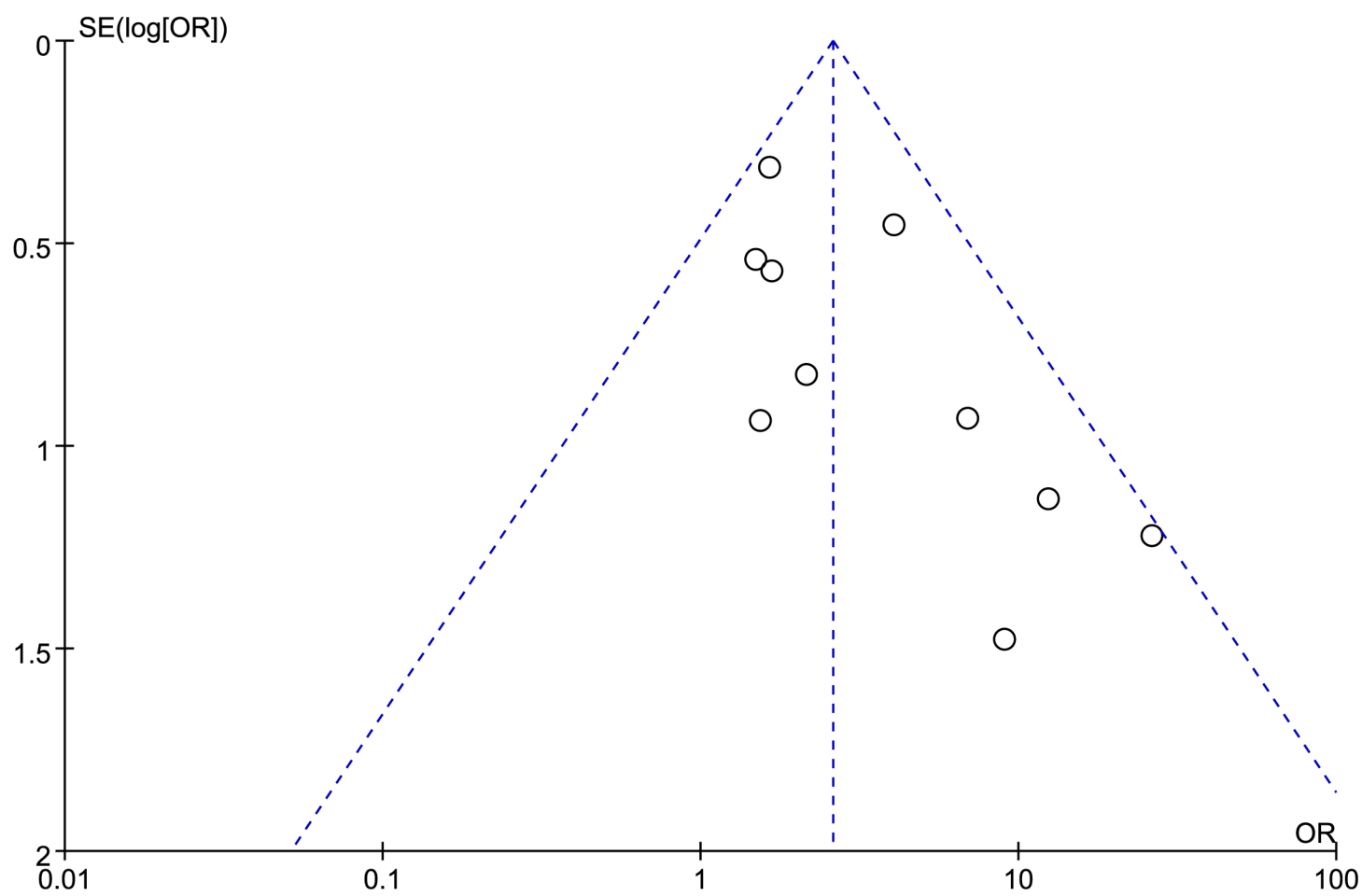

Supplement: Supplementary file 4 — Funnel plot for the 5-year mortality for STR vs BX meta-analysis. The midline of the studies indicates a slight publication bias of studies showing benefit with GTR over STR (All related studies were included). eFigure3B. Funnel plot for the 5-year mortality for STR vs BX meta-analysis. The midline of the studies indicates a slight publication bias of studies showing benefit with GTR over STR (All related studies except one high heterogeneous study were included). (PDF 2433 kb) [file 12885_2017_3909_MOESM4_ESM.pdf]

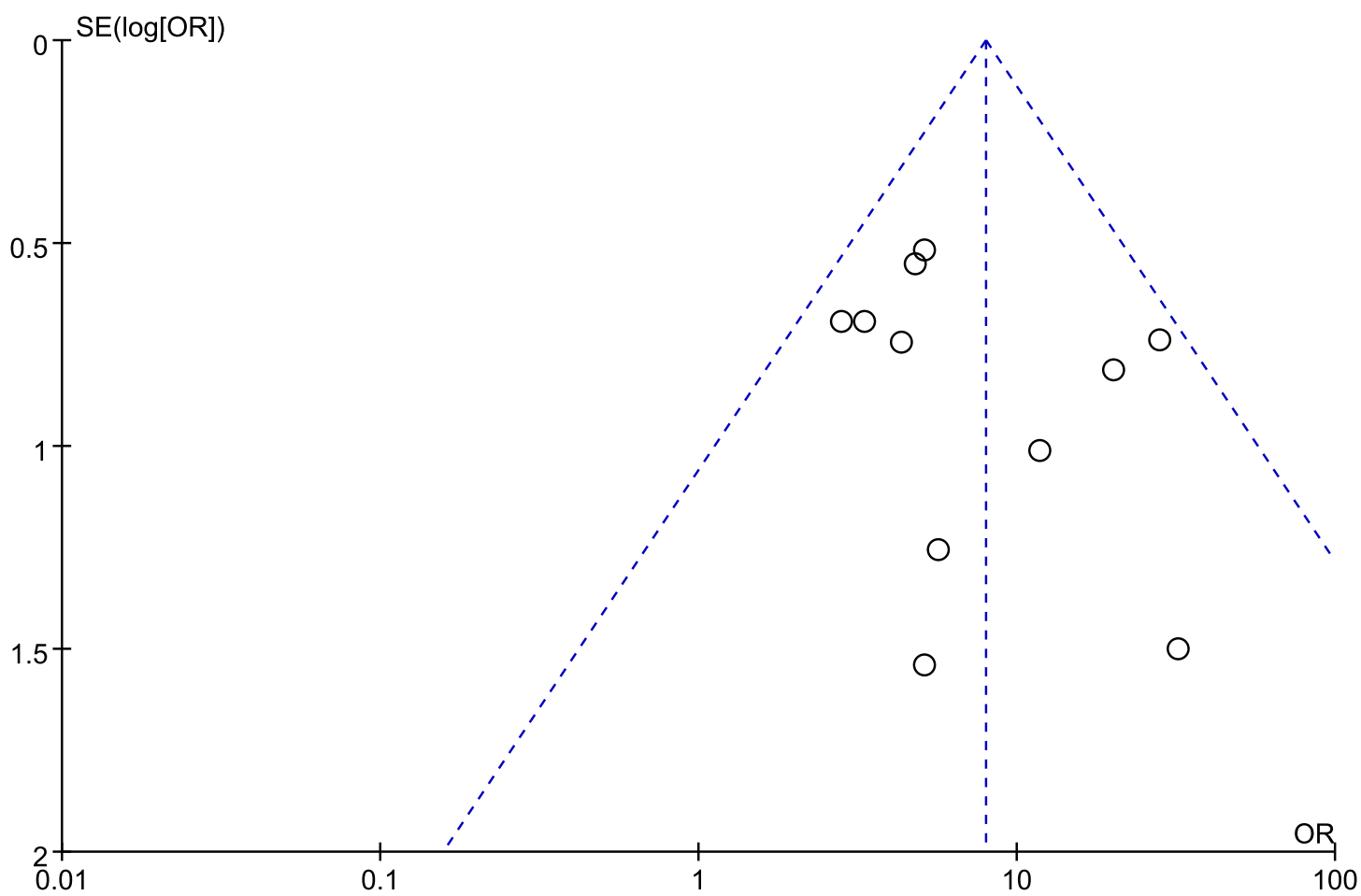

Supplement: Supplementary file 5 — Funnel plot for the 10-year mortality for GTR vs STR meta-analysis. The midline of the studies indicates a slight publication bias of studies showing benefit with GTR over STR. (PDF 12 kb) [file 12885_2017_3909_MOESM5_ESM.pdf]

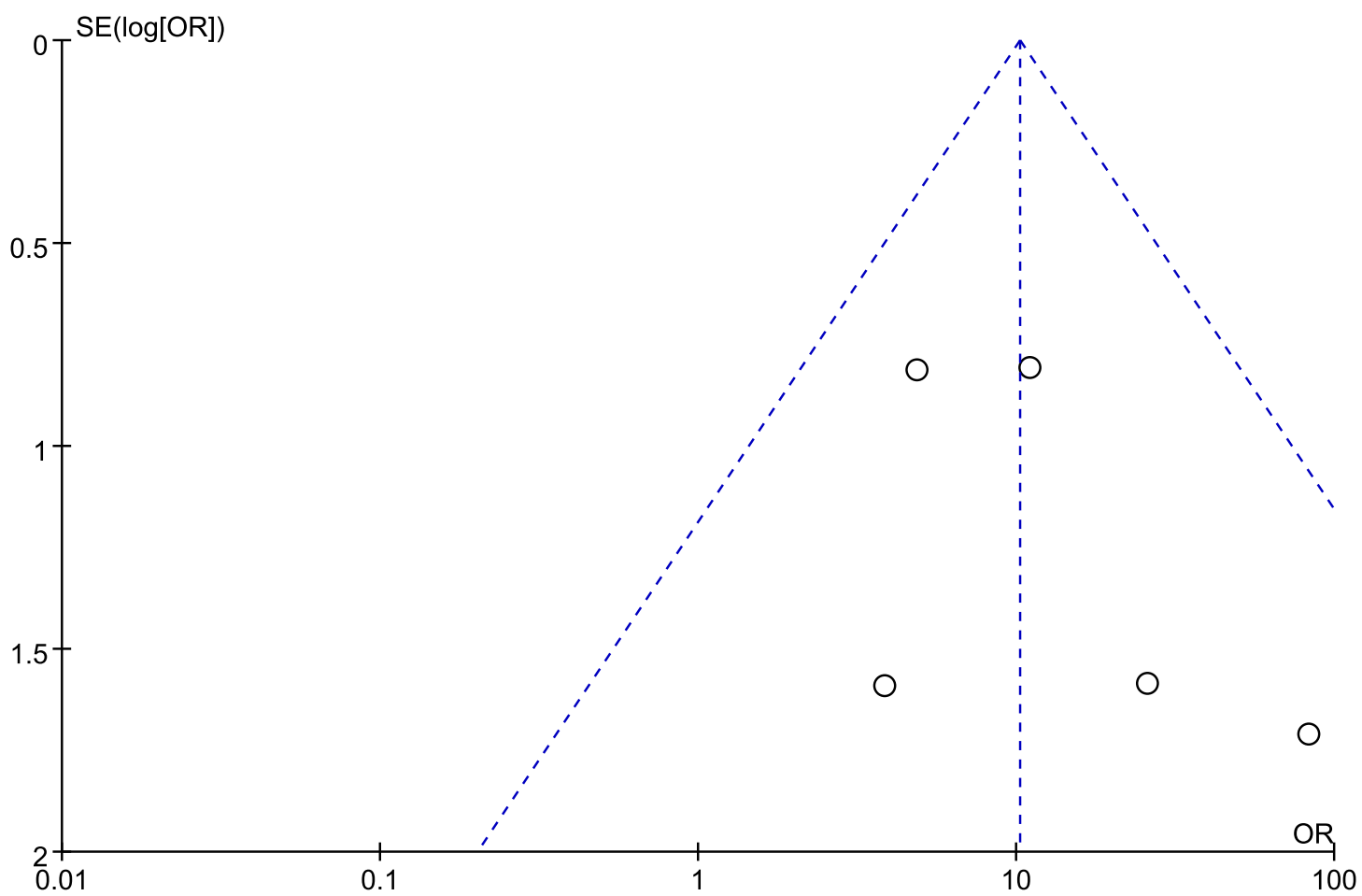

Supplement: Supplementary file 6 — Funnel plot for the 5-year mortality for GTR vs BX meta-analysis. The midline of the studies indicates a slight publication bias of studies showing benefit with GTR over STR. (PDF 12 kb) [file 12885_2017_3909_MOESM6_ESM.pdf]

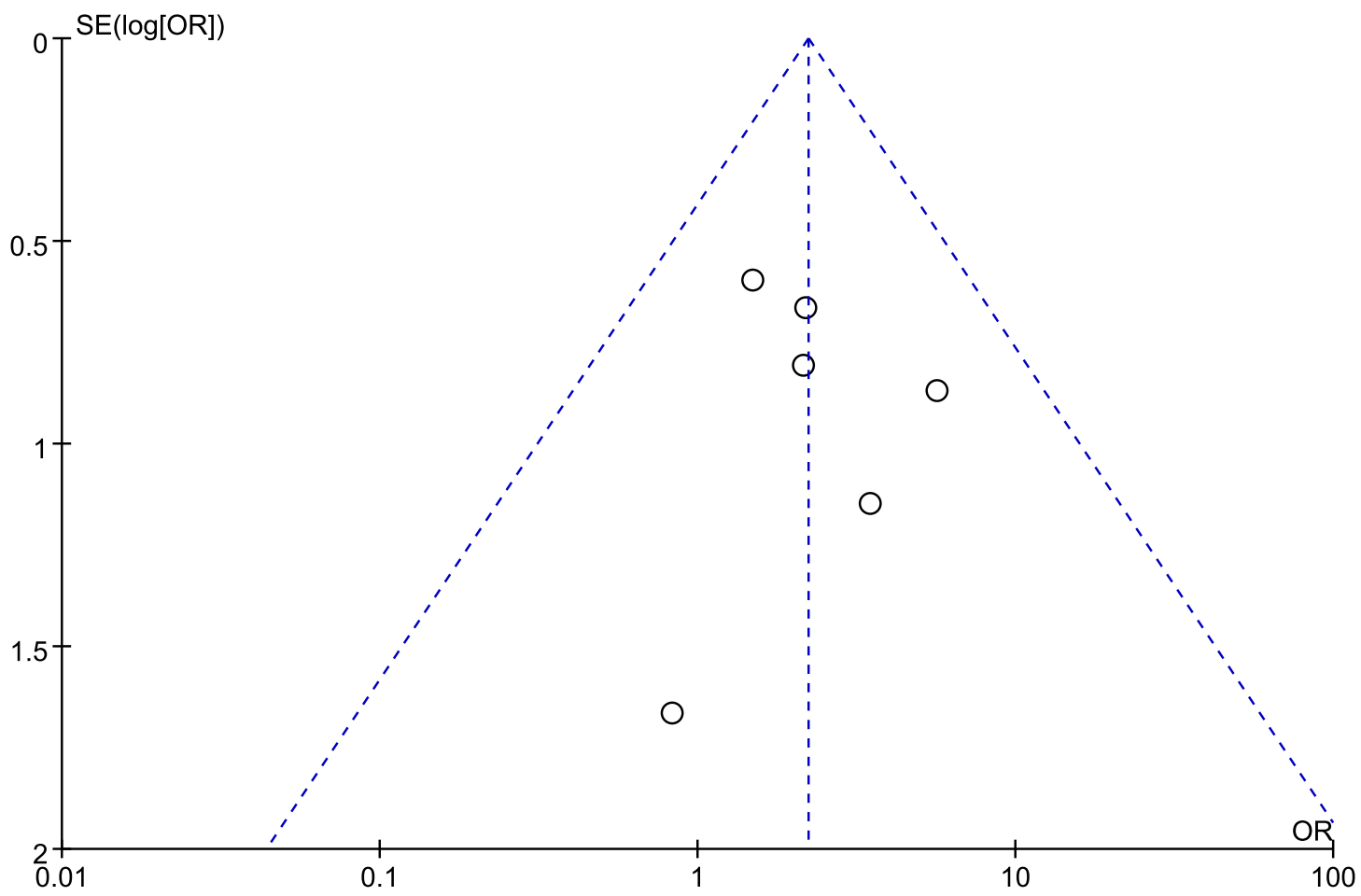

Supplement: Supplementary file 7 — Funnel plot for the 5-year mortality for STR vs BX meta-analysis. The midline of the studies indicates a slight publication bias of studies showing benefit with GTR over STR. (PDF 12 kb) [file 12885_2017_3909_MOESM7_ESM.pdf]
